# Metformin Regulating miR-34a Pathway to Inhibit Egr1 in Rat Mesangial Cells Cultured with High Glucose

**DOI:** 10.1155/2018/6462793

**Published:** 2018-02-21

**Authors:** Can Wu, Ningning Qin, Huiwen Ren, Min Yang, Shuang Liu, Qiuyue Wang

**Affiliations:** ^1^Department of Endoscope, The First Hospital Affiliated to China Medical University, Shenyang, Liaoning, China; ^2^Department of Endocrinology, The Second People's Hospital of Fuxin City, Fuxin, Liaoning, China; ^3^Department of Endocrinology, The First Hospital Affiliated to China Medical University, Shenyang, Liaoning, China

## Abstract

**Background:**

Activating AMPK*α* negatively regulates Egr1 to inhibit inflammatory cytokines in high glucose. miR-34a inhibition increases phosphorylated AMPK*α* through mediating SIRT1 to suppress the development of fatty liver.

**Aim of the Study:**

To clarify the function of Egr1 on the inflammation and fibrosis in high glucose-cultured MCs, as well as to explore the effects of metformin on miR-34a pathway and Egr1 expression.

**Methods:**

We transfected MCs with miR-34a inhibitor. And MCs were transfected with small interfering RNA for silencing Egr1 and SIRT1. Quantitative real-time PCR was used to assay the transcription levels of Egr1 mRNA and miR-34a. Western blot was used to test the protein. And ELISA was used to measure inflammatory factors.

**Results:**

High glucose upregulates Egr1 to aggravate the inflammation and fibrosis in MCs. miR-34a suppresses the activation of SIRT1/AMPK*α* and results in promoting Egr1 in high glucose-cultured MCs. Metformin attenuates high glucose-stimulated inflammation and fibrosis in MCs by regulating miR-34a-mediated SIRT1/AMPK*α* activity and the downstream Egr1 protein.

**Conclusion:**

We enriched the effects of miR-34a pathway regulating Egr1 in high glucose-cultured MCs. It provides a foundation for future researches considering Egr1 as a therapeutic target and a new direction for the clinical application of metformin in early DKD.

## 1. Introduction

Year after year, the incidence of type 2 diabetes mellitus (T2DM) increases. 20–40% of T2DM patients may develop diabetic kidney disease (DKD), which is one of the most frequent causes of end-stage renal failure. Chronic inflammation is the early pathological characteristic, including abnormal expression of inflammatory cytokines such as monocyte chemoattractant protein 1 (MCP-1) or chemokine C-X-C motif ligand 5 (CXCL5) [1]. Furthermore, high glucose may activate transforming growth factor-*β*1 (TGF-*β*1) to induce extracellular matrix (ECM) accumulation, such as increased fibronectin (FN) or connective tissue growth factor (CTGF), eventually leading to glomerular sclerosis and renal fibrosis [2].

Early growth response factor 1 (Egr1) is a zinc-finger transcription factor expressed in various cell types [3]. Recently, its expression has been shown to be rapidly upregulated in many pathological conditions, such as environmental stresses, inflammation [4], fibrosis [5], and atherogenesis [6]. Several studies have shown that the changes of Egr1 expression are linked to renal fibrosis and inflammation. Egr1 deficiency attenuated the production of proinflammatory cytokines and chemokines through the downregulation of nuclear factor *κ*B (NF-*κ*B) activity in proximal renal tubular epithelial cell (PTEC) [5]. The study by Wang et al. [4] confirmed that Egr1 activates TGF-*β*1 signaling pathway to promote high glucose-induced mesangial cells (MCs) proliferation and ECM synthesis. It is of significance in the clinic to explore the effects of Egr1 on DKD pathological mechanism.

Adenosine monophosphate-activated protein kinase *α* (AMPK*α*) activity directly inhibits Egr1 expression in hyperglycemic conditions. Valsartan inhibits angiotensin II-mediated cytokine expression via activation of AMPK*α*, which negatively regulates Egr1 in high glucose-induced macrophage cells and in aorta of streptozotocin-induced diabetic mice [7]. What is more, in high glucose conditions, the acetylation of Egr1 is increased by sirtuin 1 (SIRT1) inhibitor in murine aortic endothelial cells [6]. Interestingly, metformin reduces the inflammatory response factor NF-*κ*B expression in diabetic retinal endothelial cells by activating SIRT1/AMPK*α* pathways [8].

Metformin has been widely used in clinical glucose-lowering therapy in T2DM patients [9]. Besides, metformin has been studied more and more in the therapy of other diseases [10, 11]. In microvascular endothelial cells, metformin decreases the reactive oxygen species levels stimulated by high and the *β*-galactosidase levels related to aging [12]. Metformin inhibits renal fibrosis *in vivo* [13] and reduces the microalbuminuria in T2DM patients [14]. Meanwhile, metformin prevents liver fibrosis by downregulating miR-34a expression in nonalcoholic fatty liver disease [15].

It is well established that the expression of SIRT1 is negatively regulated by miR-34a [16]. In addition, miR-34a inhibition increases the levels of phosphorylated AMPK*α* separately through mediating PPAR*α* regulation and SIRT1 pathway to suppress the development of fatty liver [17]. Studies have shown that high glucose promotes miR-34a expression in mesangial cells (MCs). Restraining miR-34a expression can inhibit cell proliferation and relieve glomerular hypertrophy in diabetic mice [18]. However, it is not yet clear about the function and mechanism of metformin on Egr1 expression in MCs under high glucose conditions and whether miR-34a could regulate Egr1 expression via SIRT1/AMPK*α* pathways.

The aim of this study is to clarify the function of Egr1 on the inflammation and fibrosis in high glucose-cultured rat mesangial cells (RMCs) *in vitro*, as well as to explore the effects of metformin on miR-34a pathway activity and Egr1 expression.

## 2. Materials and Methods

### 2.1. Reagents and Antibodies

Metformin and 5-amino-4-imidazolecarboxamide riboside-1-b-D-ribofuranoside (AICAR) were bought from Beyotime Institute of Biotechnology (China). The antibodies used in this study include rabbit polyclonal anti-FN (H-300), rabbit polyclonal anti-SIRT1 (H-300), rabbit polyclonal anti-Egr1 (C-19), rabbit polyclonal anti-CTGF (H-55), and rabbit polyclonal anti-glyceraldehyde-3-phosphate dehydrogenase (GAPDH) (FL-335) from Santa Cruz Biotechnology (Santa Cruz, CA, USA) and rabbit monoclonal anti-AMPK*α* (4188S) and rabbit monoclonal anti-phospho-AMPK*α* (Thr172) (4811S) from Cell Signaling Technology (USA).

### 2.2. Cell Culture

We purchased rat mesangial cells (RMCs) from HBZY-1 cells, a rat mesangial cell line (China Center for Type Culture Collection, Wuhan, China). The cells were cultured in MEM medium (Life Technologies, Carlsbad, CA, USA) containing 10% fetal bovine serum (ABGENT, San Diego, CA, USA). The cells from the same passage were diluted to about 5 × 10^5^/mL and seeded in a six-well plastic plate (2 mL for each well). The cultures were incubated in a humidified atmosphere of 5% CO_2_ and 95% air at 37°C. After pre-incubation in MEM without fetal bovine serum overnight, cells were used for subsequent experiments.

### 2.3. Transient Transfection

The small interfering RNAs (siRNAs) for silencing rat SIRT1 were bought from Santa Cruz Biotechnology (10 *μ*mol/L, Santa Cruz, CA, USA). The siRNAs were for silencing rat Egr1 mRNA (GenBank number NM_012551), and the inhibitor for suppressing rat miR-34a was designed and synthesized by GeneChem (Shanghai, China), whose sequences are listed in Table 1. Reagent used in these experiments was Lipofectamine 2000 (Life Technologies, Carlsbad, CA, USA). For transient transfection, RMCs were seeded into six-well culture plates with complete medium at 30–50% confluency the day before transfection. Dilute 100 pmol siRNA or 160 pmol inhibitor in 250 *μ*L Opti-MEM® I Reduced Serum Medium without serum. Mix Lipofectamine 2000 Transfection Reagent gently before use, and then dilute 5 *μ*L in 250 *μ*L Opti-MEM I Reduced Serum Medium. Dilute oligomer and reagent in one tube, and incubate for 20 minutes at room temperature. A total of 6 hours after transfection, cells were treated with normal or high glucose media for 24 hours.

### 2.4. Isolation of MicroRNAs and miR-34a Quantitative Real-Time PCR

miRcute miRNA Isolation Kit (TianGen Biotech, Beijing, China) was used to isolate miRNAs. The primers for miR-34a were designed and synthesized by GemmaGene (Shanghai, China), whose sequences are listed in Table 2. First-strand cDNA was synthesized with a miRcute miRNA First-Strand cDNA Synthesis Kit (TianGen Biotech, Beijing, China). A miRcute miRNA qPCR Detection Kit (SYBR Green) (TianGen Biotech, Beijing, China) was used for qRT-PCR, and the program conditions included 94°C for 2 minutes and 40 cycles of 94°C for 20 seconds and 60°C for 34 seconds. Each sample was normalized to the corresponding values of the U6 control [19], whose sequences are listed in Table 2. Data analysis based on measurements of the threshold cycle was performed using the 2^−△△Ct^ method [20]. For each sample, quantitative real-time PCR was performed in triplicate.

### 2.5. RNA Extraction and Quantitative Real-Time PCR

RNA samples of RMCs were extracted by using RNAiso Plus (TaKaRa Biotechnology, Dalian, China), according to the manufacturer's instructions. The Egr1 primers were designed and synthesized by TaKaRa Biotechnology (Table 2). A total of 1 *μ*g RNA was used for reverse transcription with the PrimeScript RT Reagent Kit (TaKaRa Biotechnology, Dalian, China). A total of 2 *μ*L cDNA products was mixed with 400 nmol/L primer mixture. We used the two-step-plus-melting curve program to process all reactions in Thermal Cycler Dice Real Time System (TaKaRa Biotechnology, Dalian, China). And the data were analyzed using the ΔCt method in reference to *β*-actin. We used the 2^−△△Ct^ method [20] to analyze data based on measurements of the threshold cycle. Data are representative of three independent experiments performed in triplicate.

### 2.6. Protein Extraction and Western Blot Analysis

RMCs were seeded into 10 cm culture Petri dishes for western blot. After 0.5–24 hours, cells were harvested and lysed in RIPA lysis buffer containing PMSF protease inhibitors. Protein concentrations were determined by BCA assay (Beyotime Institute of Biotechnology, China) according to the manufacturer's recommendation. Samples were boiled at 100°C for 5 min in 5× sample buffer. Equal amounts of protein (50 *μ*g per sample) were separated by 8–12% SDS-PAGE and then transferred onto a PVDF membrane with 200 mA constant current. The blots were incubated in blocking solution, 5% BSA in Tris-buffered saline-Tween20 (TBS-T, pH 7.6), for 2 hours at room temperature. And then membranes were incubated with primary antibodies overnight at 4°C. Primary antibodies for FN, SIRT1, Egr1, AMPK*α*, phospho-AMPK*α*, and CTGF were used at a dilution ratio of 1 : 500 and normalized to GAPDH, which was used at a dilution ratio of 1 : 1000. PVDF membranes were treated with anti-rabbit (1 : 10000) secondary antibodies for 1 hour at room temperature. We used MicroChemi 4.2 Bio-imaging system to detect immunoreactive bands and used Gelpro32 software to analyze the relative grey value. Data are representative of three independent experiments.

### 2.7. Assay for Cell Supernatant MCP-1 and CXCL5 Concentrations

Cell culture supernatant samples were centrifuged for 10 min (6000 r/min, 4°C). MCP-1 (product number: ELR-MCP1, RayBiotech, Norcross, GA, USA) commercial sandwich ELISA Kit was used to test MCP-1 protein levels. CXCL5 (product number: KA1828, Abnova, Taiwan) commercial sandwich ELISA Kit was used to measure CXCL5 protein levels.

### 2.8. Statistical Analyses

All data, normalized to baseline or control activity in individual experiment, were assessed by IBM SPSS statistical software package (v.19.0, IBM Corp., USA, 2010). Statistical significance was analyzed by ANOVA, followed by LSD's test for normally distributed values. And the data were expressed as means ± SDs. All *p* values reported were two-tailed, and a *p* value of <0.05 was considered statistically significant, whereas <0.001 was highly significant.

We followed the methods of Wu et al. [21].

## 3. Results

### 3.1. High Glucose-Induced Higher Expression of Egr1 mRNA and Protein in RMCs

**Figure 1:**
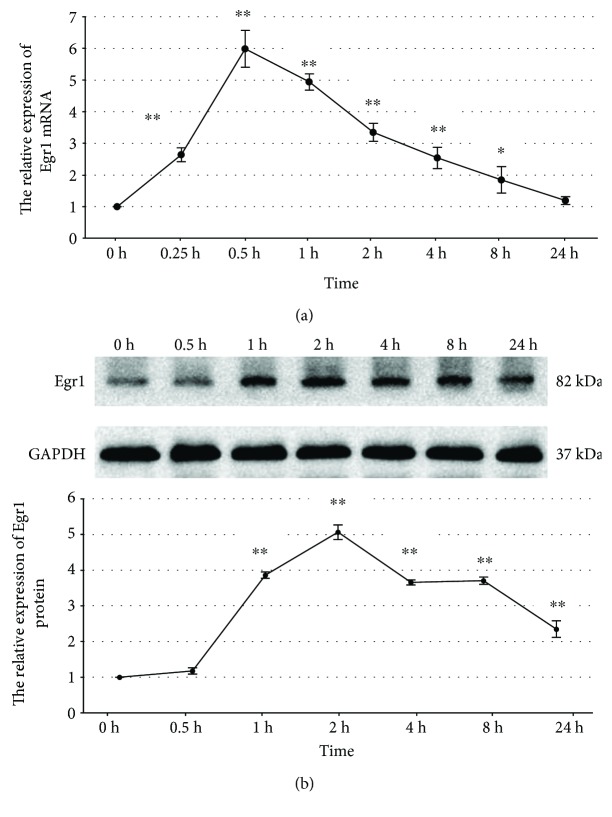
The effects of high glucose on Egr1 mRNA (a) and protein (b) expression. RMCs were incubated with high glucose (30 mmol/L glucose) for 0–24 hours. mRNA expression was determined by quantitative real-time PCR, and protein expression was determined by western blotting. ^∗^*p* < 0.05, ^∗∗^*p* < 0.001 versus time zero control. All results represent means ± SD obtained from three independent experiments in triplicate. Egr1: early growth response factor 1; GAPDH: glyceraldehyde-3-phosphate dehydrogenase.

**Figure 2:**
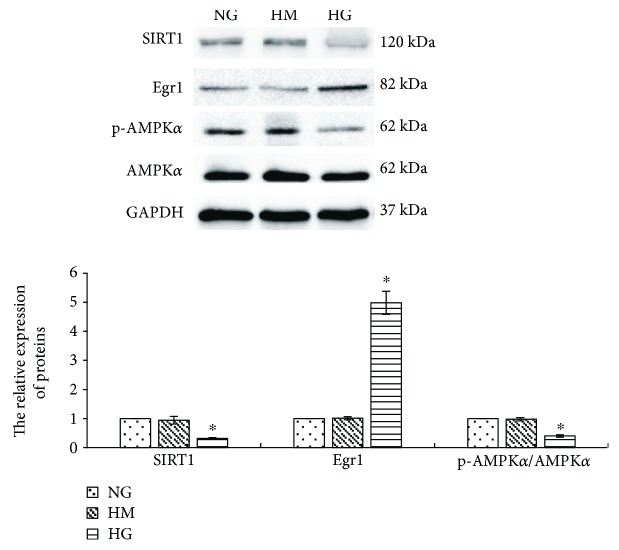
High glucose obviously suppresses SIRT1 protein and the p-AMPK*α*/AMPK*α* ratio (by western blotting) in RMCs and increases Egr1 protein (by western blotting) in RMCs. RMCs were incubated with normal glucose (NG, 5.5 mmol/L glucose), high mannitol (HM, 5.5 mmol/L glucose + 24.5 mmol/L mannitol), or high glucose (HG, 30 mmol/L glucose). ^∗^*p* < 0.001 versus NG control. All results represent means ± SD obtained from three independent experiments in triplicate and normalized to GAPDH. SIRT1: sirtuin 1; Egr1: early growth response factor 1; p-AMPK*α*: phosphorylated adenosine monophosphate-activated protein kinase *α*; AMPK*α*: adenosine monophosphate-activated protein kinase *α*; GAPDH: glyceraldehyde-3-phosphate dehydrogenase.

To clarify the effects of high glucose on Egr1 expression in MCs, we used a rat mesangial cell line (HBZY-1 cells) [4]. Incubation of RMCs with high glucose (30 mmol/L) for 24 hours showed time-dependent upregulation of Egr1 expression. Egr1 mRNA transcript levels had a peak stimulation of 5.98-fold after 30 minutes of exposure (*p* < 0.001), which returned towards the baseline at 24 hours (Figure 1(a)). The Egr1 protein expression levels determined by western blotting revealed similar temporal patterns, with a peak stimulation of 5.06-fold after 2 hours of exposure (*p* < 0.001) (Figure 1(b)). And then RMCs treated with high mannitol (24.5 mmol/L) serve as an osmotic control. We cultured RMCs under conditions of normal glucose (5.5 mmol/L), high mannitol, or high glucose, respectively, for 2 hours. Western blotting assays of Egr1 protein revealed a significantly higher expression in high glucose conditions compared with normal glucose (*p* < 0.001) (Figure 2). There was no statistically significant difference between normal glucose and high mannitol conditions (Figure 2), so we eliminated the influence of osmotic pressure.

### 3.2. Activating AMPK*α* Suppresses High Glucose-Induced Egr1 Expression in RMCs

It has been demonstrated that AMPK*α* activity plays a certain protective effect on diabetic kidney [22]. Activating AMPK*α* negatively regulated Egr1, which led to inhibition of high glucose-induced inflammatory cytokine in monocytic cells [7].

Firstly, we incubated RMCs under conditions of normal glucose, high mannitol, or high glucose, respectively, for 24 hours. Results determined by western blotting revealed that high glucose obviously reduced AMPK*α* phosphorylation (*p* < 0.001) (Figure 2). Whereas there was no statistically significant difference under normal glucose and high mannitol conditions (Figure 2), we eliminated the influence of osmotic pressure.

**Figure 3:**
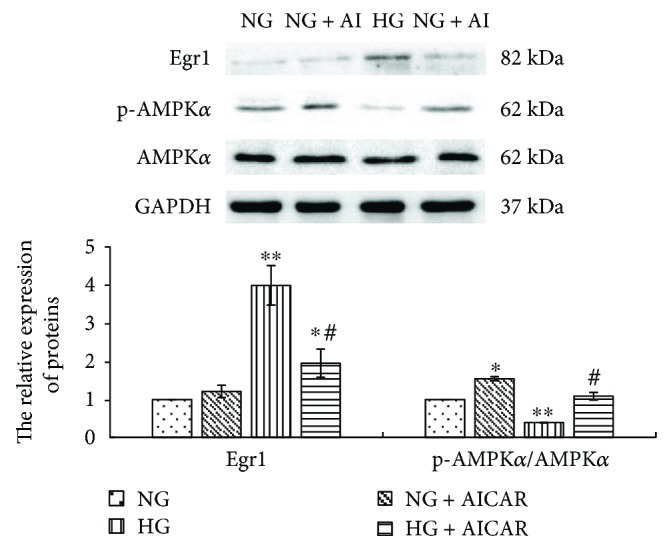
Activating AMPK*α* significantly reduces Egr1 expression (by western blotting) stimulated by high glucose in RMCs. RMCs were pre-incubated with 1 mmol/L AMPK*α* activator AICAR (+AI) for 30 minutes before stimulating with normal glucose (NG, 5.5 mmol/L glucose) or high glucose (HG, 30 mmol/L glucose). ^∗^*p* < 0.05, ^∗∗^*p* < 0.001 versus RMCs cultured with normal glucose media. ^#^*p* < 0.001 versus RMCs cultured with high glucose media. All results represent means ± SD obtained from three independent experiments in triplicate and normalized to GAPDH. AICAR: 5-amino-4-imidazolecarboxamide riboside-1-b-D-ribofuranoside; Egr1: early growth response factor 1; p-AMPK*α*: phosphorylated adenosine monophosphate-activated protein kinase *α*; AMPK*α*: adenosine monophosphate-activated protein kinase *α*; GAPDH: glyceraldehyde-3-phosphate dehydrogenase.

To explore the effects of AMPK*α* on Egr1 generation, RMCs were pretreated with 1 mmol/L AMPK*α* activator AICAR for 30 minutes prior to high glucose and incubated for 2 hours for detection of Egr1 and 24 hours for detection of AMPK*α*. Western blotting assays of AMPK*α* phosphorylation revealed that AICAR increased phosphorylated AMPK*α* (*p* < 0.05) (Figure 3). The higher expression of Egr1 protein induced by high glucose was dramatically reduced by AICAR (*p* < 0.001) (Figure 3). This suggests that activating AMPK*α* prevents the higher expression of Egr1 protein induced by high glucose.

### 3.3. High Glucose Upregulates miR-34a Expression in RMCs

**Figure 4:**
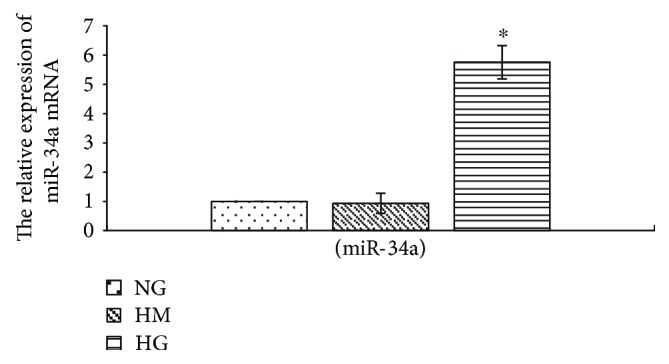
High glucose upregulates miR-34a expression. RMCs were incubated with normal glucose (NG, 5.5 mmol/L glucose), high mannitol (HM, 5.5 mmol/L glucose + 24.5 mmol/L mannitol), or high glucose (HG, 30 mmol/L glucose) for 24 hours. ^∗^*p* < 0.001 versus RMCs cultured with normal glucose media. mRNA expression was determined by quantitative real-time PCR. All results represent means ± SD obtained from three independent experiments in triplicate.

To analyze the effects of high glucose on miR-34a expression in RMCs, we treated RMCs with normal glucose, high mannitol, or high glucose, respectively, for 24 hours. The results, examined by qReal-time PCR, indicated that high glucose raised miR-34a expression when compared to normal glucose (*p* < 0.001) (Figure 4). Whereas there was no statistically significant difference between normal glucose and high mannitol conditions in miR-34a expression (Figure 4), we eliminated the influence of osmotic pressure.

### 3.4. High Glucose Suppresses the Activation of SIRT1/AMPK*α* via Inducing miR-34a Higher Expression in RMCs

miR-34a regulates the development of obesity and age-related diseases via inhibiting SIRT1 expression [23]. In addition, miR-34a regulates AMPK*α* activity through mediating SIRT1 pathway to suppress the development of fatty liver [17].

At first, RMCs were cultured with conditions of normal glucose, high mannitol, or high glucose, respectively, for 24 hours. We found that high glucose significantly decreased SIRT1 protein expression in RMCs (*p* < 0.001) (Figure 2). Whereas there was no statistically significant difference under normal glucose and high mannitol conditions (Figure 2), we eliminated the influence of osmotic pressure.

**Figure 5:**
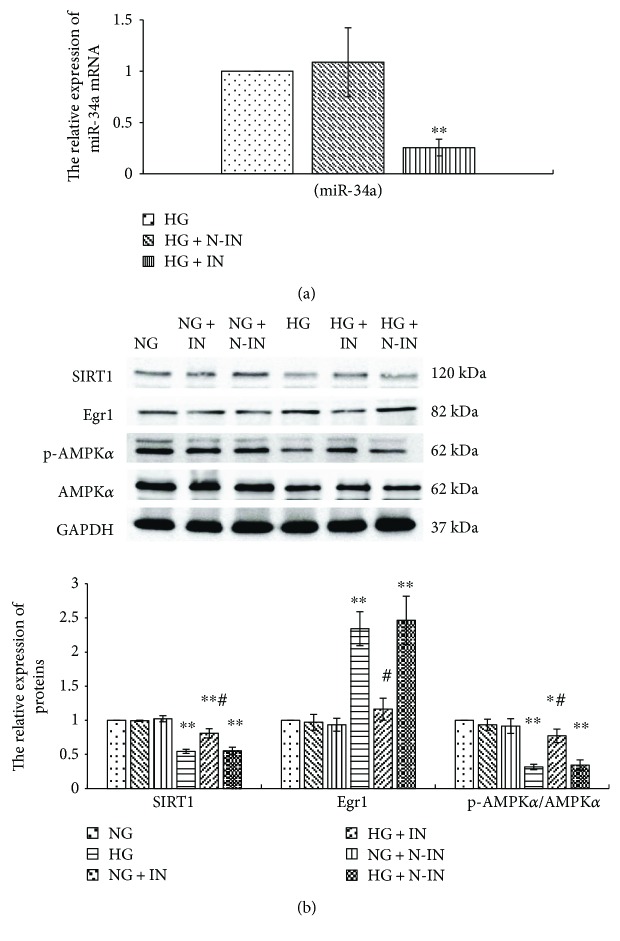
miR-34a inhibitor significantly suppresses miR-34a expression (by quantitative real-time PCR) stimulated by high glucose (a). And miR-34a inhibitor activates SIRT1/AMPK*α* and restrains Egr1 expression (by western blotting) in high glucose conditions (b) in RMCs. RMCs were transfected with miR-34a inhibitor (IN) or nonspecific control (N-NC) for 6 hours and further cultured with normal glucose (NG, 5.5 mmol/L glucose) or high glucose (HG, 30 mmol/L glucose) media for 24 hours. ^∗^*p* < 0.05, ^∗∗^*p* < 0.001 versus RMCs cultured with normal glucose media. ^#^*p* < 0.001 versus RMCs cultured with high glucose media. All results represent means ± SD obtained from three independent experiments in triplicate. And western blotting results were normalized to GAPDH. SIRT1: sirtuin 1; Egr1: early growth response factor 1; p-AMPK*α*: phosphorylated adenosine monophosphate-activated protein kinase *α*; AMPK*α*: adenosine monophosphate-activated protein kinase *α*; GAPDH: glyceraldehyde-3-phosphate dehydrogenase.

To confirm the role of miR-34a on regulating the activation of SIRT1/AMPK*α* pathways in high glucose conditions, we transfected RMCs with miR-34a inhibitor, which had a reduction of 74.51% in miR-34a mRNA expression (*p* < 0.001) (Figure 5(a)). The effects of high glucose on suppressing the activation of SIRT1/AMPK*α* pathways were significantly reversed in miR-34a inhibitor-transfected cells (*p* < 0.001) (Figure 5(b)).

**Figure 6:**
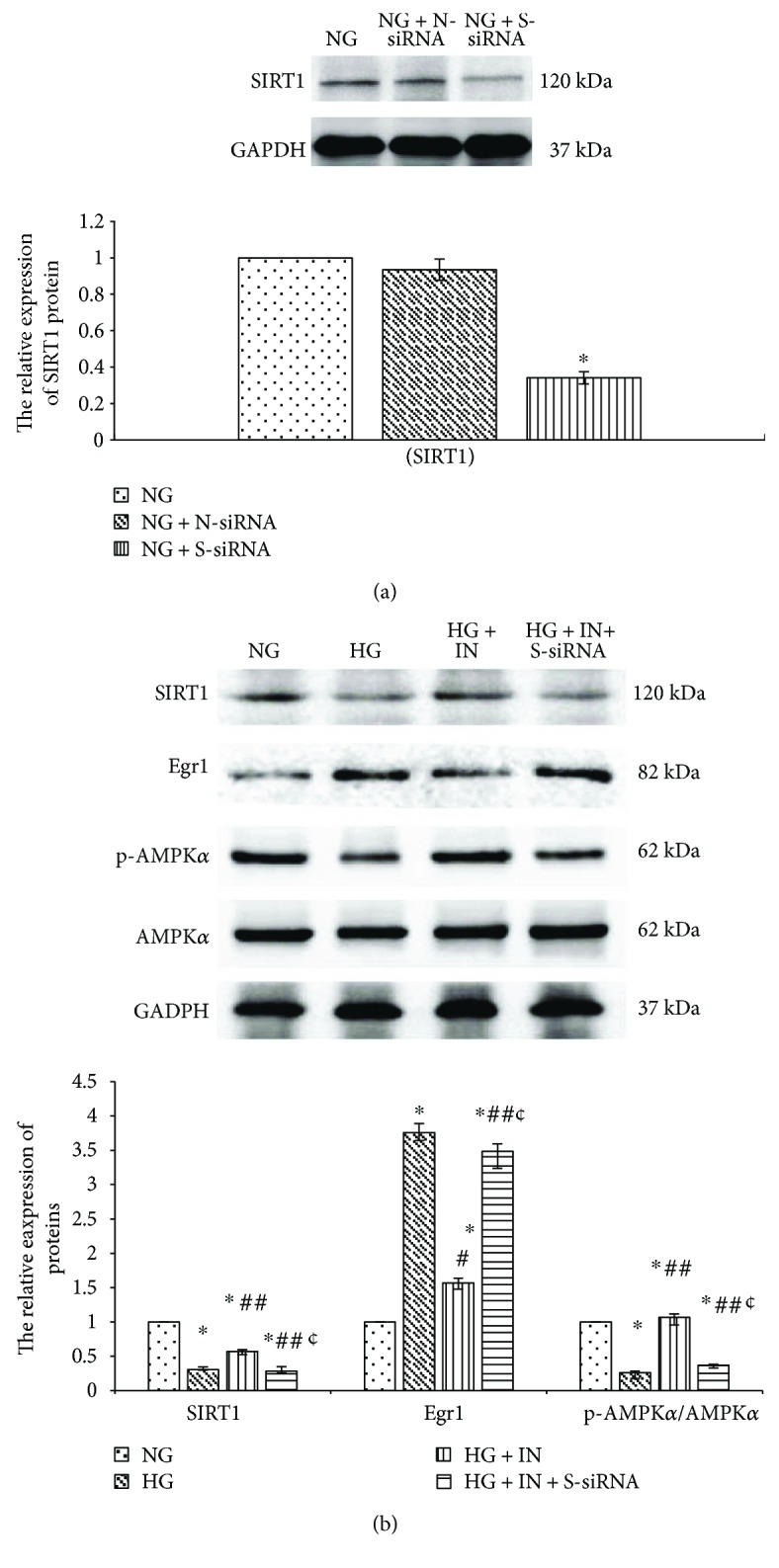
SIRT1 gene silencing suppresses SIRT1 protein (by western blotting) expression in RMCs (a). Transfecting with SIRT1-siRNA obviously decreases phosphorylated AMPK*α* and increases Egr1 expression (by western blotting) though miR-34a inhibitor in high glucose-cultured RMCs (b). RMCs were transfected with SIRT1-siRNA (S-siRNA), nonspecific control (N-siRNA), or miR-34a inhibitor (IN) for 6 hours and further cultured with normal glucose (NG, 5.5 mmol/L glucose) or high glucose (HG, 30 mmol/L glucose) media for 24 hours. ^∗^*p* < 0.001 versus RMCs cultured with normal glucose media. ^#^*p* < 0.05, ^##^*p* < 0.001 versus RMCs cultured with high glucose media. ^¢^*p* < 0.001 versus RMCs transfected with miR-34a inhibitor. All results represent means ± SD obtained from three independent experiments in triplicate and normalized to GAPDH. SIRT1: sirtuin 1; Egr1: early growth response factor 1; p-AMPK*α*: phosphorylated adenosine monophosphate-activated protein kinase *α*; AMPK*α*: adenosine monophosphate-activated protein kinase *α*; GAPDH: glyceraldehyde-3-phosphate dehydrogenase.

Based on transfecting with miR-34a inhibitor, we treated the RMCs with SIRT1-siRNA, which suppressed SIRT1 protein expression with a reduction of 65.91% (*p* < 0.001) (Figure 6(a)). SIRT1-siRNA obviously decreased phosphorylated AMPK*α* though RMCs had been transfected with miR-34a inhibitor in high glucose conditions (*p* < 0.001) (Figure 6(b)). This suggests that suppressing miR-34a expression reverses the inhibitory effects of high glucose on AMPK*α* activation via promoting SIRT1 expression.

### 3.5. miR-34a Upregulates Egr1 Expression via Suppressing the Activation of SIRT1/AMPK*α* in High Glucose-Cultured RMCs

Our research has demonstrated that high glucose-stimulated miR-34a higher expression. Inhibiting miR-34a transcript reversed the suppressing effects of high glucose on the activation of SIRT1/AMPK*α* pathways. Activating AMPK*α* decreased high glucose-stimulated Egr1 expression. Therefore, we further explored whether miR-34a regulates Egr1 expression via regulating the activation of SIRT1/AMPK*α* pathways.

Firstly, we transfected RMCs with miR-34a inhibitor for 6 hours and then cultured with high glucose for 24 hours. The results examined by western blot revealed that miR-34a inhibitor dramatically reduced the high glucose-stimulated Egr1 protein expression (*p* < 0.001), while it had no effect on the basal levels under normal glucose (Figure 5(b)). This suggests that inhibition of miR-34a gene expression could suppress Egr1 expression induced by high glucose in RMCs.

What is more, RMCs were transfected with miR-34a inhibitor as well as SIRT1-siRNA, then exposed for 24 hours to high glucose. SIRT1-siRNA significantly increased Egr1 expression though RMCs had been transfected with miR-34a inhibitor in high glucose conditions (*p* < 0.001) (Figure 6(b)). This suggests that miR-34a inhibitor could mediate Egr1 overexpression via activating SIRT1/AMPK*α* pathways.

### 3.6. High Glucose Upregulates the Expression of Fibrosis Factors and Inflammation Factors via Promoting Egr1 Expression in RMCs

**Figure 7:**
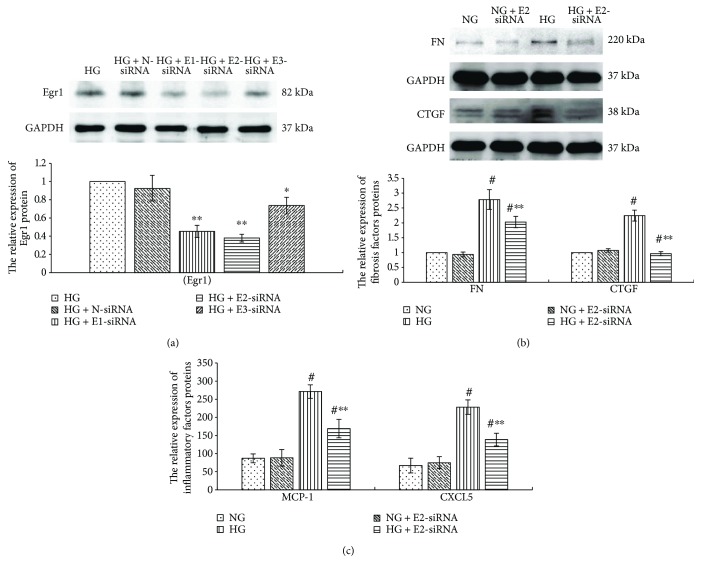
Egr1 gene silencing suppresses the Egr1 protein expression (by western blotting) stimulated by high glucose (a). Egr1 gene silencing suppresses the upregulated expression of fibrosis factors (FN and CTGF) (b) (by western blotting) and inflammatory factors (MCP-1 and CXCL5) (c) (by ELISA) stimulated by high glucose in RMCs. RMCs were transfected with nonspecific siRNA (N-siRNA) or three different siRNA targeting Egr1 gene (E1-siRNA, E2-siRNA, and E3-siRNA) for 6 hours and then cultured with high glucose media (30 mmol/L glucose) or normal glucose (NG, 5.5 mmol/L glucose) for 24 hours. ^∗^*p* < 0.05, ^∗∗^*p* < 0.001 versus RMCs cultured with high glucose media. ^#^*p* < 0.001 versus RMCs cultured with normal glucose media. All results represent means ± SD obtained from three independent experiments in triplicate and normalized to GAPDH. Egr1: early growth response factor 1; FN: fibronectin; CTGF: connective tissue growth factor; GAPDH: glyceraldehyde-3-phosphate dehydrogenase; MCP-1: monocyte chemoattractant protein 1; CXCL5: chemokine C-X-C motif ligand 5.

Inflammation reaction and fibrosis are the main features of early DKD [1, 24]. To confirm the effects of Egr1 on inflammation and fibrosis in high glucose-cultured RMCs, we suppressed Egr1 expression by transfecting RMCs with Egr1-siRNA. RMCs were transfected with three different Egr1-siRNAs (E1-siRNA, E2-siRNA, and E3-siRNA) targeting Egr1 gene or nonspecific siRNA (N-siRNA) for 6 hours and then cultured with high glucose for 24 hours. Compared with high glucose control group, transfection with three different Egr1-siRNA decreased Egr1 expression. And E2-siRNA was the most efficient, with reduction of 62.05% in the Egr1 protein expression (*p* < 0.001) (Figure 7(a)). We therefore used E2-siRNA in subsequent experiments. Whereas there was no statistically significant difference between the HG and N-siRNA groups in the Egr1 protein expression (Figure 7(a)), we eliminated the influence of transfection.

As shown in Figure 7(b), the results revealed that E2-siRNA dramatically reduced the high glucose-stimulated expression of fibrosis factors (FN and CTGF) (*p* < 0.001), and inflammation factors (MCP-1 and CXCL5) (*p* < 0.001), whereas it had no effect on their basal expression levels under normal glucose. This suggests that silencing Egr1 expression prevents the higher expression of fibrosis and inflammation factors induced by high glucose in RMCs.

### 3.7. Metformin Suppresses High Glucose Stimulation of the Expression of Fibrosis and Inflammation Factors

Metformin inhibits renal fibrosis *in vivo* [13], and it reduces the microalbuminuria in T2DM patients. In this study, we further explored the function and mechanism of metformin on the fibrosis and inflammation stimulated by high glucose in RMCs.

**Figure 8:**
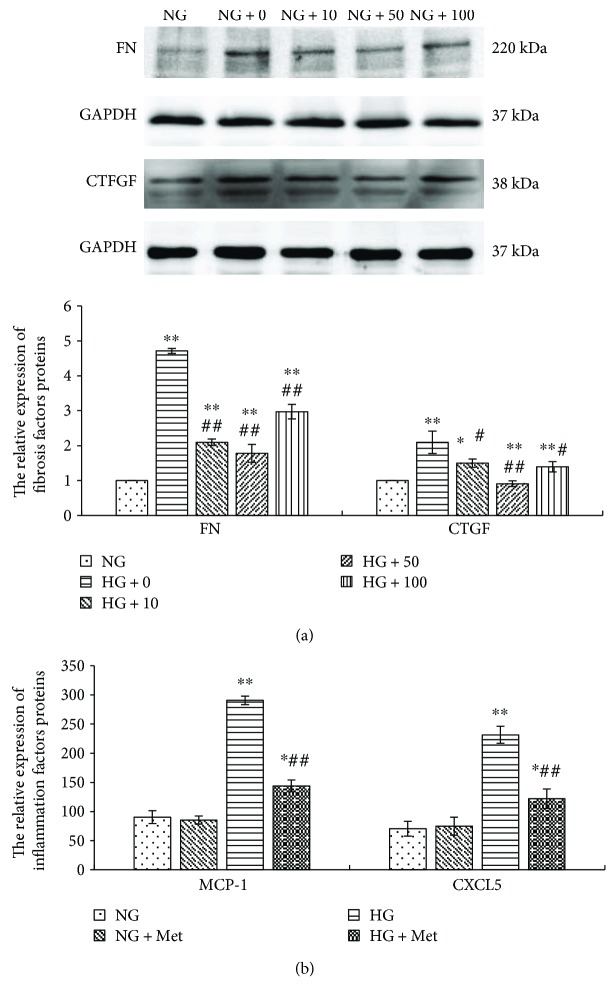
Different concentration of metformin (10, 50, and 100 *μ*mol/L) significantly reduces the expression of fibrosis factors (FN and CTGF) (by western blotting) stimulated by high glucose in RMCs, and 50 *μ*mol/L is the most efficient (a). 50 *μ*mol/L metformin dramatically reduces the high glucose-stimulated expression of inflammatory factors (MCP-1 and CXCL5) (by ELISA) in RMCs (b). RMCs were pre-incubated with metformin for 1 hour before stimulating with normal glucose (NG, 5.5 mmol/L glucose) or high glucose (HG, 30 mmol/L glucose) for 24 hours. ^∗^*p* < 0.05, ^∗∗^*p* < 0.001 versus RMCs cultured with normal glucose media. ^#^*p* < 0.05, ^##^*p* < 0.001 versus RMCs cultured with high glucose media. All results represent means ± SD obtained from three independent experiments in triplicate. And western blotting results were normalized to GAPDH. Met: metformin; FN: fibronectin; CTGF: connective tissue growth factor; GAPDH: glyceraldehyde-3-phosphate dehydrogenase; MCP-1: monocyte chemoattractant protein 1; CXCL5: chemokine C-X-C motif ligand 5.

Firstly, RMCs were incubated with different concentrations of metformin for 1 hour prior to high glucose treatment for 24 hours. We observed that different concentrations of metformin (10, 50, and 100 *μ*mol/L) significantly reduced the expression of FN and CTGF stimulated by high glucose (*p* < 0.05). Additionally, 50 *μ*mol/L metformin was the most efficient, with a reduction of 62.14% in FN (*p* < 0.001) and 56.15% in CTGF (*p* < 0.001) protein expression when compared with high glucose (Figure 8). We therefore pretreated RMCs with metformin at a concentration of 50 *μ*mol/L for 1 hour and then grew them in normal or high glucose for 24 hours. The results were determined by ELISA, the expression of MCP-1 and CXCL5 stimulated by high glucose was dramatically reduced by metformin pretreatment (*p* < 0.001), whereas there was no effect on their basal expression levels under normal glucose (Figure 8). This suggests that metformin restrains the high glucose-stimulated expression of fibrosis and inflammation factors in RMCs.

### 3.8. Metformin Prevents High Glucose-Induced miR-34a Expression in RMCs

**Figure 9:**
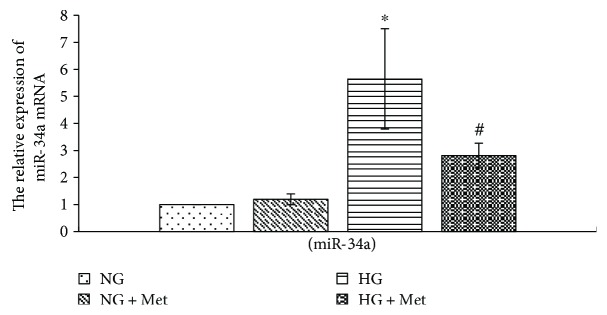
Metformin prevents high glucose-induced miR-34a expression in RMCs. RMCs were pre-incubated with 50 *μ*mol/L metformin for 1 hour before stimulating with normal glucose (NG, 5.5 mmol/L glucose) or high glucose (HG, 30 mmol/L glucose) for 24 hours. ^∗^*p* < 0.001 versus RMCs cultured with normal glucose media. ^#^*p* < 0.001 versus RMCs cultured with high glucose media. mRNA expression was determined by quantitative real-time PCR. All results represent means ± SD obtained from three independent experiments in triplicate.

There are studies that discovered that metformin downregulates miR-34a expression in nonalcoholic fatty liver disease and inhibits liver fibrosis [15]. In this study, we demonstrated that high glucose obviously increases miR-34a expression in RMCs. We then explored whether metformin regulated miR-34a expression in high glucose-cultured RMCs. We pretreated RMCs with 50 *μ*mol/L metformin for 1 hour prior to normal or high glucose treatment for 24 hours. The results, examined by qReal-time PCR, indicated that miR-34a expression in RMCs pretreated with metformin was obviously lower than high glucose group (*p* < 0.001) (Figure 9). This suggests that metformin restrains high glucose-stimulated miR-34a expression.

### 3.9. Metformin Activates SIRT1/AMPK*α* Pathways in High Glucose-Cultured RMCs

**Figure 10:**
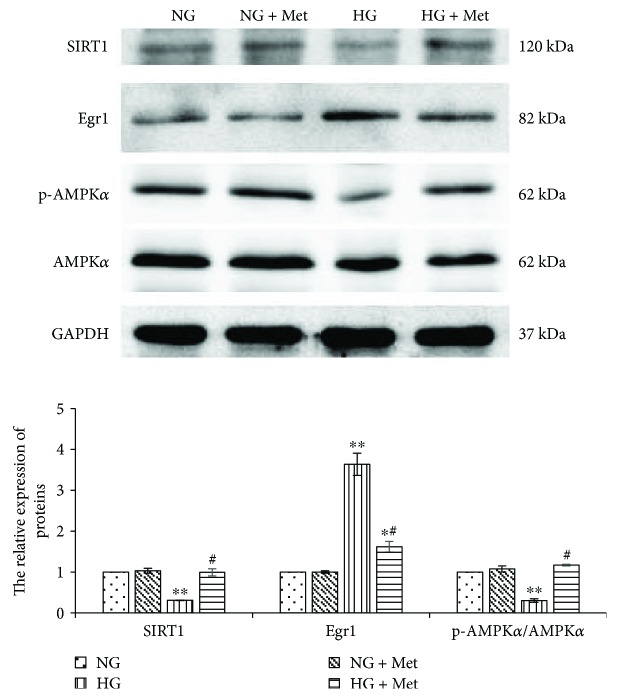
Metformin reverses the high glucose downregulated SIRT1/AMPK*α* and upregulated Egr1 protein expression (by western blotting) in RMCs. RMCs were pre-incubated with 50 *μ*mol/L metformin for 1 hour before stimulating with normal glucose (NG, 5.5 mmol/L glucose) or high glucose (HG, 30 mmol/L glucose) for 24 hours. ^∗^*p* < 0.05, ^∗∗^*p* < 0.001 versus RMCs cultured with normal glucose media. ^#^*p* < 0.001 versus RMCs cultured with high glucose media. All results represent means ± SD obtained from three independent experiments in triplicate and normalized to GAPDH. Met: metformin; SIRT1: sirtuin 1; Egr1: early growth response factor 1; p-AMPK*α*: phosphorylated adenosine monophosphate-activated protein kinase *α*; AMPK*α*: adenosine monophosphate-activated protein kinase *α*; GAPDH: glyceraldehyde-3-phosphate dehydrogenase.

To investigate the effects of metformin on SIRT1/AMPK*α* activity the downstream pathways of miR-34a, we pretreated RMCs with 50 *μ*mol/L metformin for 1 hour prior to normal or high glucose treatment for 24 hours. We discovered that the protein expression of SIRT1 in metformin pretreatment group was obviously higher than that in high glucose group (*p* < 0.001). Meanwhile, the results also revealed that metformin pretreatment significantly increased the pAMPK/AMPK*α* ratio compared with high glucose (*p* < 0.001) (Figure 10). This suggests that, in high glucose-cultured RMCs, metformin not only reverses the SIRT1 protein expression, but also activates AMPK*α*.

### 3.10. Metformin Prevents High Glucose-Induced Egr1 Expression in RMCs

We explored whether metformin regulated Egr1 the downstream of SIRT1/AMPK*α* pathways. We pretreated RMCs with 50 *μ*mol/L metformin for 1 hour prior to normal or high glucose treatment for 2 hours. The results, examined by western blotting, indicated that the Egr1 protein expression in RMCs pretreated with metformin was obviously lower than high glucose group (*p* < 0.001) (Figure 10). This suggests that metformin downregulates the Egr1 protein expression stimulated by high glucose in RMCs.

## 4. Discussion

Metformin has been a first-line treatment for T2DM since the 1850s [9]. Due to its superior safety and relatively low risk of side effects, metformin has been tested for its effectiveness in the treatment of other diseases. Recent studies have demonstrated that metformin has obvious therapeutic effects in cancer and cardiovascular [10, 11]. Moreover, it can prevent renal fibrosis [13] and the inflammatory response in diabetic microvascular endothelial cells [8].

Both of ECM accumulation [25, 26] and inflammatory response [1, 24] in glomerular mesangial cells are the early pathological features of DKD. Our study confirms that high glucose stimulates inflammatory factors (MCP-1 and CXCL5) and fibrosis factors (FN and CTGF) expression. When we treated high glucose-cultured MCs with metformin, the results revealed that the expression of inflammation and fibrosis cytokines declined. And metformin at a concentration of 50 *μ*mol/L has a maximum inhibition effect. It prompts that metformin can weaken the inflammation and fibrosis in high glucose-cultured MCs. The concentration in our study has a certain clinical significance according to the pharmacokinetics of metformin [27].

Egr1, a zinc-finger transcription factor of the immediate early gene family, plays a role in regulation of inflammation in cholestatic liver injury [28], ischemic and reperfusion lung injury [29], and atherogenesis [6]. High glucose rapidly upregulates Egr1 expression in glomerular endothelial cells [30], renal tubular epithelial cells [5], aortic endothelial cells [6], and renal cortical fibroblasts [30]. In this study, the results revealed that the expression of Egr1 increased in MCs under 30 mmol/L glucose, compared with that of normal glucose. The mRNA transcription levels of Egr1 peaked after 30 minutes of exposure to high glucose, while the Egr1 protein expression peaked after 2 hours of exposure. This is consistent with the report by Wang et al. [4] providing evidence that high glucose-induced transcription factor Egr1 expression in MCs. Meanwhile, they confirmed that Egr1 was upregulated in kidney tissue from 40-week-old diabetes rats [4].

Egr1 aggravates renal failure via facilitating NF-*κ*B-mediated renal innate immunity [5]. Suppressing Egr1 activity inhibits renal interstitial fibrosis via downregulation of TGF-*β*, *α*-smooth muscle actin (*α*SMA), and type I collagen [31]. We extended to explore the effects of Egr1 on MCs in high glucose medium. MCs were transfected with Egr1-siRNA and incubated under high glucose conditions; the expression levels of fibrosis and inflammatory cytokines were lower than that of MCs transfected with the nonspecific siRNA. Similarly, the study by Wang et al. [4] confirmed that Egr1 upregulates ECM synthesis in MCs under high glucose. These results suggest that high glucose upregulates Egr1 to aggravate the inflammation and fibrosis in MCs.

As a kind of small RNA nonencoded in the body, miRNAs can regulate target gene expression at the transcription levels [32]. Studies have found that miRNAs could regulate a variety of mechanism to affect diabetes development and may play certain roles in the pathogenesis of DKD [33, 34]. High glucose can promote miR-34a overexpression in MCs. Moreover, downregulating miR-34a expression inhibits cell proliferation and then alleviates glomerular hypertrophy in diabetic mice [18].

We have demonstrated that the expression of miR-34a increased under high glucose in MCs in this study. It is well established that SIRT1 is a direct target of miR-34a [16]. Some scholars have found that the inflammatory reactions were attenuated by activating SIRT1/AMPK*α* signaling pathways in diabetic retinal endothelial cells [8]. We also found that MCs treated with high glucose significantly decreased SIRT1 protein generation. Meanwhile, high glucose reduces the activation of AMPK*α* by phosphorylation of a threonine residue (Thr^172^) in RMCs.

We transfected MCs with miR-34a inhibitor to clarify the effects of miR-34a on regulating the activity of SIRT1/AMPK*α* in high glucose environment. miR-34a inhibitor attenuates the negative effects of high glucose on the expression of SIRT1 protein in RMCs. As well, miR-34a inhibitor reverses the negative effects of high glucose on AMPK*α* activity. It should be noted that treatment with siRNA-SIRT1 prevents the activating effects of miR-34a inhibitor on AMPK*α* in high glucose-stimulated MCs. In previous study, Ding et al. [17] have found a similar result: miR-34a inhibition increases the levels of phosphorylated AMPK*α* separately through mediating PPAR*α* regulation and SIRT1 pathway in hepatic steatosis mice. These results suggest that miR-34a suppresses AMPK*α* phosphorylation via downregulating SIRT1 in high glucose-cultured MCs.

Study has found that AMPK*α* activity directly inhibits Egr1 expression in hyperglycemic conditions [7]. We treated MCs with AMPK*α* activator AICAR prior to high glucose. Results revealed that AICAR obviously downregulates high glucose-stimulated Egr1 expression. Moreover, inhibition of SIRT1 activity via aldose reductase (AR) causes increased acetylation and prolonged expression of Egr1 leading to proinflammatory and prothrombotic responses in diabetic atherosclerosis [6]. We have drawn inferences that miR-34a suppresses AMPK*α* phosphorylation via downregulating SIRT1 in high glucose-cultured MCs. Then we extended to clear whether miR-34a indirectly regulates Egr1 expression via adjusting the activity of SIRT1/AMPK*α* signaling pathways.

Treatment with miR-34a inhibitor restrained the Egr1 expression stimulated by high glucose in MCs. In addition, the inhibition of miR-34a inhibitor on Egr1 expression can be restored when we transfected MCs with SIRT1-siRNA in high glucose medium. We did not find Egr1 is a direct target protein of miR-34a according to TargetScan Release 5.0. We presumed that miR-34a might indirectly promote Egr1-mediated inflammation and fibrosis via suppressing the activation of SIRT1/AMPK*α* in high glucose-cultured RMCs. However, the specific mechanism of miR-34a regulating Egr1 activity still needs more researches to elucidate.

What is more, metformin downregulates miR-34a expression in nonalcoholic fatty liver disease and results in preventing liver fibrosis [15]. And then we treated MCs with metformin prior to high glucose. As a result, the high glucose-stimulated miR-34a expression was significantly reduced after metformin treatment. What is more, treatment with metformin resulted in activating SIRT1/AMPK*α* pathways and significantly reduced Egr1 in high glucose-cultured MCs. We speculated that metformin might promote SIRT1/AMPK*α* activity and downregulate the downstream Egr1 protein via suppressing miR-34a in high glucose-stimulated MCs. It suggests that metformin might alleviate the inflammation and fibrosis in high glucose-stimulated MCs via regulating miR-34a-mediated SIRT1/AMPK*α* activity and the downstream Egr1 protein expression. However, more detailed researches are needed in order to further explore the specific mechanism of metformin regulating miR-34a expression.

At present, the clinical application of metformin is prudent in diabetic patients with mild renal insufficiency [35]. Our study indicates that metformin prevents high glucose-stimulated fibrosis and inflammatory cytokines in MCs. It promotes that metformin might provide protection against diabetic kidney injury. Metformin might also have a certain inhibitory effect on inflammation and fibrosis in early DKD. However, our study lacks evidence in animal model *in vivo* experiments. It still needs more detailed researches to elucidate whether the beneficial effect of metformin on miR-34a expression is direct or indirect and to explore the possible mechanism. It is of great significance to elucidate whether the clinical application of metformin is relaxed restrictions on renal function.

## 5. Conclusion

**Figure 11:**
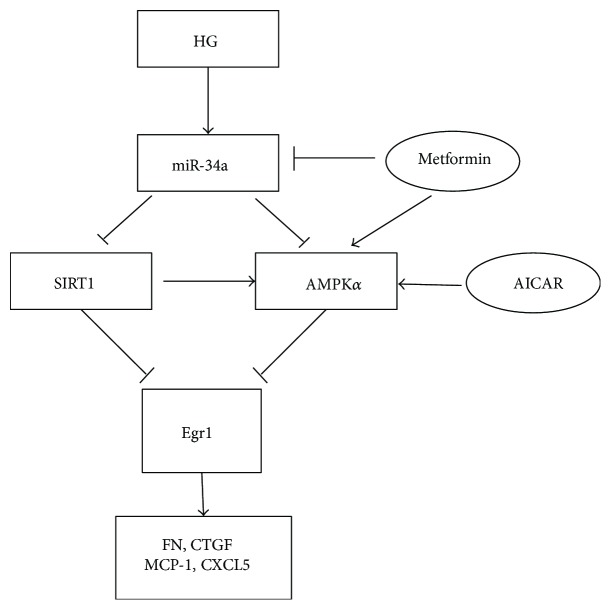
Schematic representation of miR-34a regulating SIRT1/AMPK*α* signaling pathways and Egr1 involved in the HG-induced fibrosis and inflammation in RMCs. High glucose upregulates miR-34a and Egr1 expression, as well as decreases SIRT1 protein and AMPK*α* phosphorylation expression. miR-34a suppresses the activation of SIRT1/AMPK*α* and results in promoting Egr1-mediated inflammation and fibrosis in high glucose-cultured RMCs. AICAR activating AMPK*α* prevents Egr1 expression in high glucose. Meanwhile, metformin attenuates high glucose-stimulated inflammation and fibrosis in RMCs by regulating miR-34a-mediated SIRT1/AMPK*α* activity and the downstream Egr1 protein. HG: high glucose; SIRT1: sirtuin 1; AMPK*α*: adenosine monophosphate-activated protein kinase *α*; AICAR: 5-amino-4-imidazolecarboxamide riboside-1-b-D-ribofuranoside; Egr1: early growth response factor 1; FN: fibronectin; CTGF: connective tissue growth factor; MCP-1: monocyte chemoattractant protein 1; CXCL5: chemokine C-X-C motif ligand 5; RMCs: rat mesangial cells.

Consequently, our experimental results show that miR-34a suppresses the activation of SIRT1/AMPK*α* and results in promoting Egr1-mediated inflammation and fibrosis in high glucose-cultured RMCs. Meanwhile, metformin attenuates high glucose-stimulated inflammation and fibrosis in RMCs by regulating miR-34a-mediated SIRT1/AMPK*α* activity and the downstream Egr1 protein (Figure 11).

Based on the abovementioned analysis, our study preliminary discusses the mechanism of metformin attenuating high glucose-stimulated inflammation and fibrosis at the cellular level. And we enriched the effects of miR-34a pathways regulating Egr1 expression in high glucose-cultured MCs. Thus, we believe our findings provide a new theoretical foundation to elucidate the molecular mechanism of metformin regulating the DKD development. It provides a foundation for future researches considering Egr1 as a therapeutic target and a new direction for the clinical application of metformin in early DKD.

## Figures and Tables

**Table 1:** The gene sequences used for transfection.

Gene	Sequences	
Egr1-siRNA	E1-siRNA sense	5′-CCA ACA GUG GCA ACA CUU UTT-3′
	Antisense	5′-AAA GUG UUG CCA CUG UUG GTT-3′
	E2-siRNA sense	5′-CCA GUC CCA ACU CAU CAA ATT-3′
	Antisense	5′-UUU GAU GAG UUG GGA CUG GTT-3′
	E3-siRNA sense	5′-GGA CAA GAA AGC AGA CAA ATT-3′
	Antisense	5′-UUU GUC UGC UUU CUU GUC CTT-3′
	N-siRNA sense	5′-UUC UCC GAA CGU GUC ACG UTT-3′
	Antisense	5′-ACG UGA CAC GUU CGG AGA ATT-3′

miR-34a inhibitor	Inhibitor	5′-ACA ACC AGC UAA GAC ACU GCC A-3′
	Inhibitor-NC	5′-CAG UAC UUU UGU GUA CAA-3′

**Table 2:** Primer sequences used for real-time PCR primers.

Primer	Sequences	
Egr1	Forward	5′-CAC CAG ACC ATG CTT CAG TGA GA-3′
	Reverse	5′-GTT GCA TGG CTG TTC ACA GGA-3′

*β*-Actin	Forward	5′-TGA CAG GAT GCA GAA GGA GAT TAC-3′
	Reverse	5′-GAG CCA ATC CAC ACA GA-3′

miR-34a	Forward	5′-AGC CGC TGG CAG TGT CTT A-3′
	Reverse	5′-CAG AGC AGG GTC CGA GGT A-3′

U6	Forward	5′-CGA GCU GGU AAA GAA UUU ATT-3′
	Reverse	5′-UAA AUU CUU UAC CAG CUC GTT-3′
